# Contrasting structures of the Southern Benue trough and the contiguous crystalline basement as observed from high-resolution aeromagnetic data

**DOI:** 10.1038/s41598-023-48639-8

**Published:** 2023-12-06

**Authors:** Leke Sunday Adebiyi, Akinola Bolaji Eluwole, Akindeji Opeyemi Fajana, Naheem Banji Salawu, Aliyu Saleh

**Affiliations:** 1 Department of Physical Sciences, Landmark University, PMB 1001, Omu-Aran, Kwara State Nigeria; 2 Department of Geophysics, Federal University, Oye-Ekiti, Ekiti State Nigeria; 3 BS Geophysical and Consultancy Ltd, Ilorin, Nigeria; 4 Physics Unit, School of Preliminary and Continuing Education, Ibrahim Badamasi Babangida University, Lapai, Nigeria

**Keywords:** Solid Earth sciences, Geophysics

## Abstract

The present study investigated crustal structures and geological bodies within selected areas of the southern Benue trough and the adjacent crystalline basement. It utilized high-resolution aeromagnetic data to provide original insights into the contrasting structures and geological bodies within the area, while also identifying promising areas for further mineral and hydrocarbon investigations. The aeromagnetic anomaly data were analyzed using various techniques, including total gradient magnitude, pseudo-gravity transformation, tilt derivative, and source parameter imaging. The total gradient magnitude anomalies revealed a considerable component of the basin affected by block faulting which may have resulted from severe tectonic and structural deformation of the metamorphic basement and the subsequent injection into the sedimentary sequence, a few intrusions. The pseudo-gravity transformation identified a large igneous body in the sedimentary basin which is characterized by positive pseudo-gravity anomalies and surrounded on both sides by linear negative anomalies that signify a rift zone. The positive amplitude of the tilt derivative identified subtle linear magnetic minerals associated with the geologic structure and igneous bodies that suggest considerable mineral exploration prospects. A two-dimensional model of the source parameter imaging reveals the basement below the sedimentary basin to be intensely fragmented and variably subsided with the floor of the basin forming irregular upward and downward folds. In conclusion, the findings suggest promising prospects for mineral exploration along the margin of the sedimentary basin and potential hydrocarbon resources in the northeastern segment of the basin, which is characterized by fewer intrusions.

## Introduction

**Figure 1:**
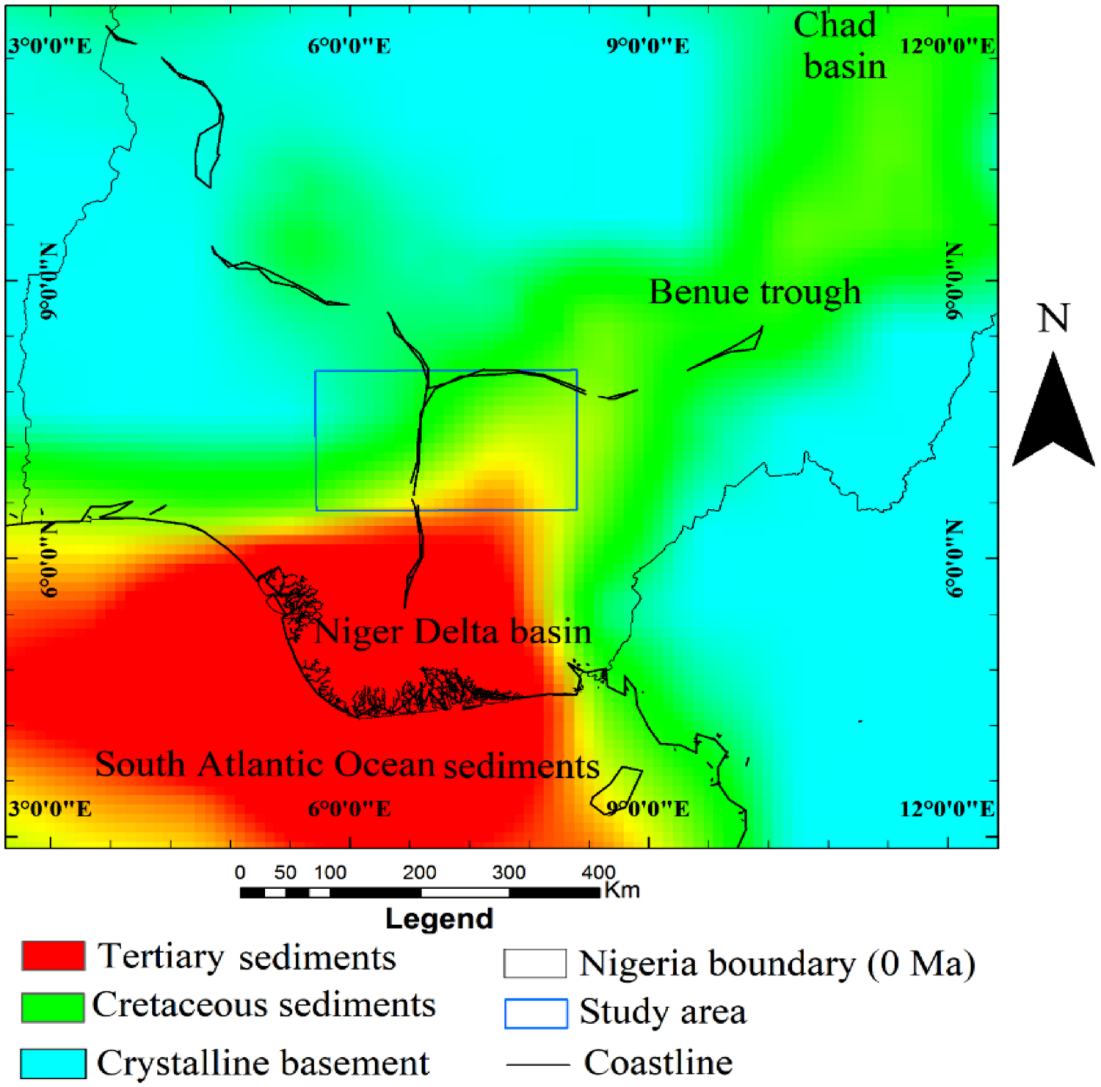
Reconstructed map of Nigeria at 0 Ma showing the boundary of the Benue trough, Niger Delta basin and the crystalline basement.

The Nigerian Benue trough is an intra-continental rift basin during the Cretaceous period^1–6^. The trough continues inland from the petroleum-bearing Niger Delta basin and stretches nearly 800 km to northeastern Nigeria, where it bifurcates into the northern (Gongola) and the southern (Yola) arms. Figure 1 is a reconstructed map showing the present-day (0 Ma) boundaries of the Benue trough; the Niger Delta basin (a protraction of the South Atlantic marine sediments) and the crystalline basement of Nigeria^7,8^. The southern arm of the Benue trough is connected to a deep depression beneath the southern boundary of the Chad basin while the northern arm continues below the Chad basin as a protracted depression that stretches much further than Lake Chad^1–3,9^. The Nigerian Benue trough is seemingly divided into the Lower, Middle and Upper parts with no observable geological boundaries. It has widths between 130 and 150 km^6^.

Magnetic data have played major roles in understanding and characterizing geological formations around the world^10–14^. The data is used frequently to delineate magnetic thin sheets or contacts such as faults. This has been used frequently in petroleum exploration where magnetic data is used to identify faults in the basement that may have control over the depositional history of the sedimentary basin^15–19^. In this case, the interpreter must have an understanding of the structural and lithological framework and the importance of regional deposition patterns^20–24^.

Several groundbreaking studies have been conducted in the Benue trough, many of which have significantly enhanced our understanding of this geological environment. Pioneering studies by authors such as^1,25–32^ consistently reveal a distinctive central axial positive gravity anomaly, flanked by elongated negative gravity anomalies.

Reference^33^ analysed airborne magnetic data in the lower and middle Benue trough and unveiled several igneous rocks within the sedimentary sequence and the crystalline basement. These rocks are characterized by varying thicknesses and magnetization polarities, and together with the basement, exhibit depths ranging from 1.7 to 7.30 km.

Reference^3^ promulgated a geological model for the origin of the Benue trough during the Lower Cretaceous. Notable findings include the formation of isolated basins in the Aptian, the development of a substantial delta in the upper Benue trough during the Albian, and a Turonian transgression connecting Atlantic and Tethys waters through the Sahara, Niger basins, and Benue trough. The proposed tectonic evolution involves transcurrent faulting, resulting in compressional and tensional regimes, with major compressional phases occurring during the Santonian and at the end of the Cretaceous. The study suggested sinistral wrenching along the trough during the initial stages of the opening of the Gulf of Guinea.

Additional key findings by many authors including^1,2,5,30,34,35^ have significantly advanced our knowledge. Despite these collective efforts, certain knowledge gaps persist, including the use of low-resolution geophysical data, insufficient data to consolidate the trough development in relation to plate drifting, and a predominant focus on the Lower Cretaceous to the end of the Cretaceous, leading to a gap in understanding more recent geological features and potential post-Cretaceous changes.

However, recent studies by many authors, including^4,6,36–47^ have provided additional insights into the geological features and potential hydrocarbon and mineral resources by analysing one, two, or all of airborne magnetic data; gravity data; and satellite data in various parts of the Benue trough. Despite these advancements, critical gaps persist including the absence of an up-to-date review of the trough development in relation to plate movement, as well as an inept description of the contrasting features of the Cretaceous basin and the adjoining crystalline basement.

### Location and geologic setting

**Figure 2:**
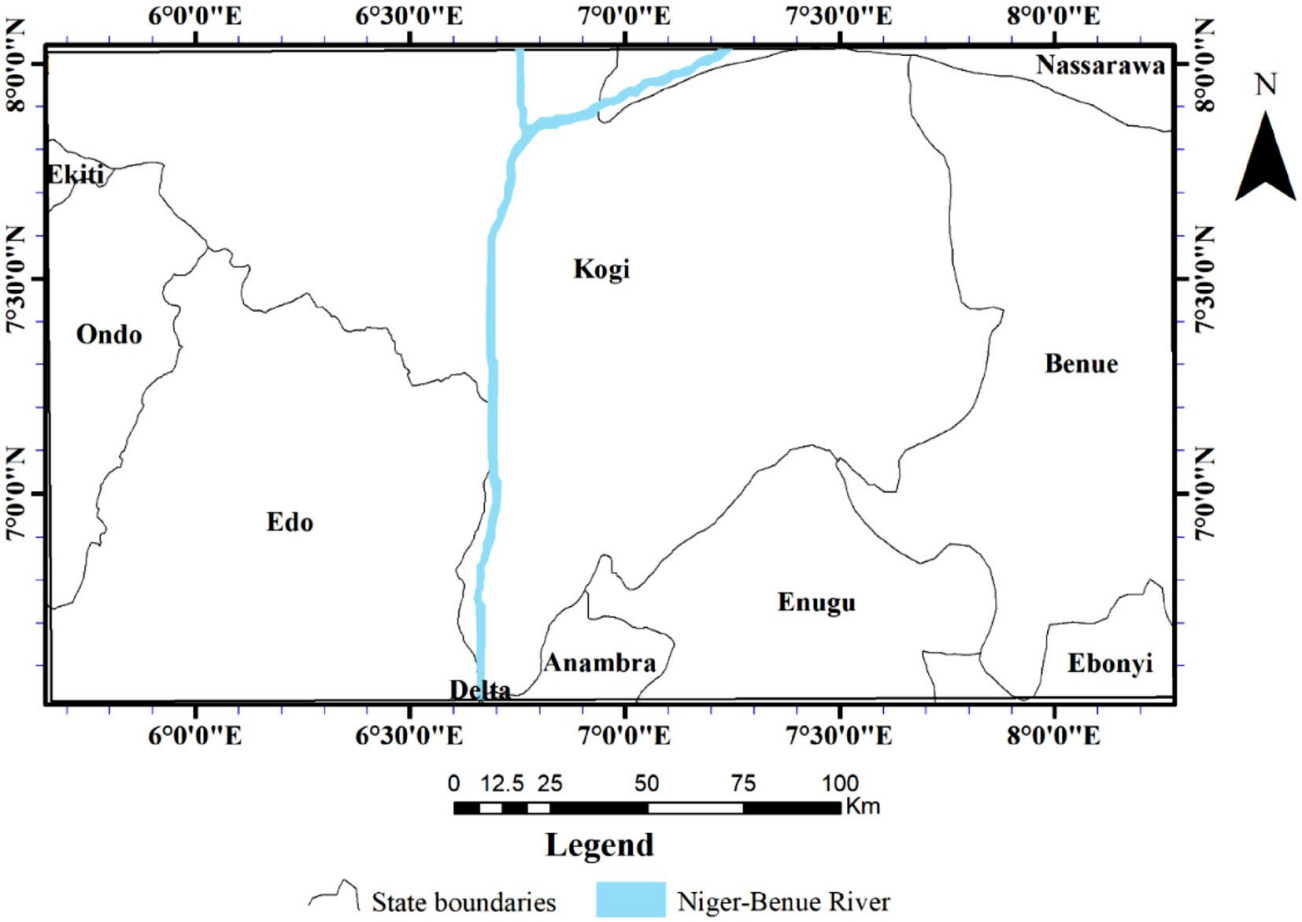
Location map showing the Nigerian provinces that fall within the study area.

The study area extends from longitude 5°30′ $E$ to 8°30′ $E$ and latitude 6°00′ $N$ to $8^\circ {00}^{\mathrm{^{\prime}}}N$ encompassing parts of Benue, Kogi, Edo, Enugu, and Ondo, as well as small portions of Nasarawa, Anambra, Ebonyi and Ekiti States in the southern parts of Nigeria (Fig. 2). It is underlain by the Cretaceous sediments of the southern Benue trough and the crystalline basement of southwestern Nigeria. The Cretaceous sediments in the study area extend slightly downward, intersecting with the Tertiary sediments of the Niger Delta basin, and westward into the crystalline basement of southwestern Nigeria.

### Sedimentary terrane

**Figure 3:**
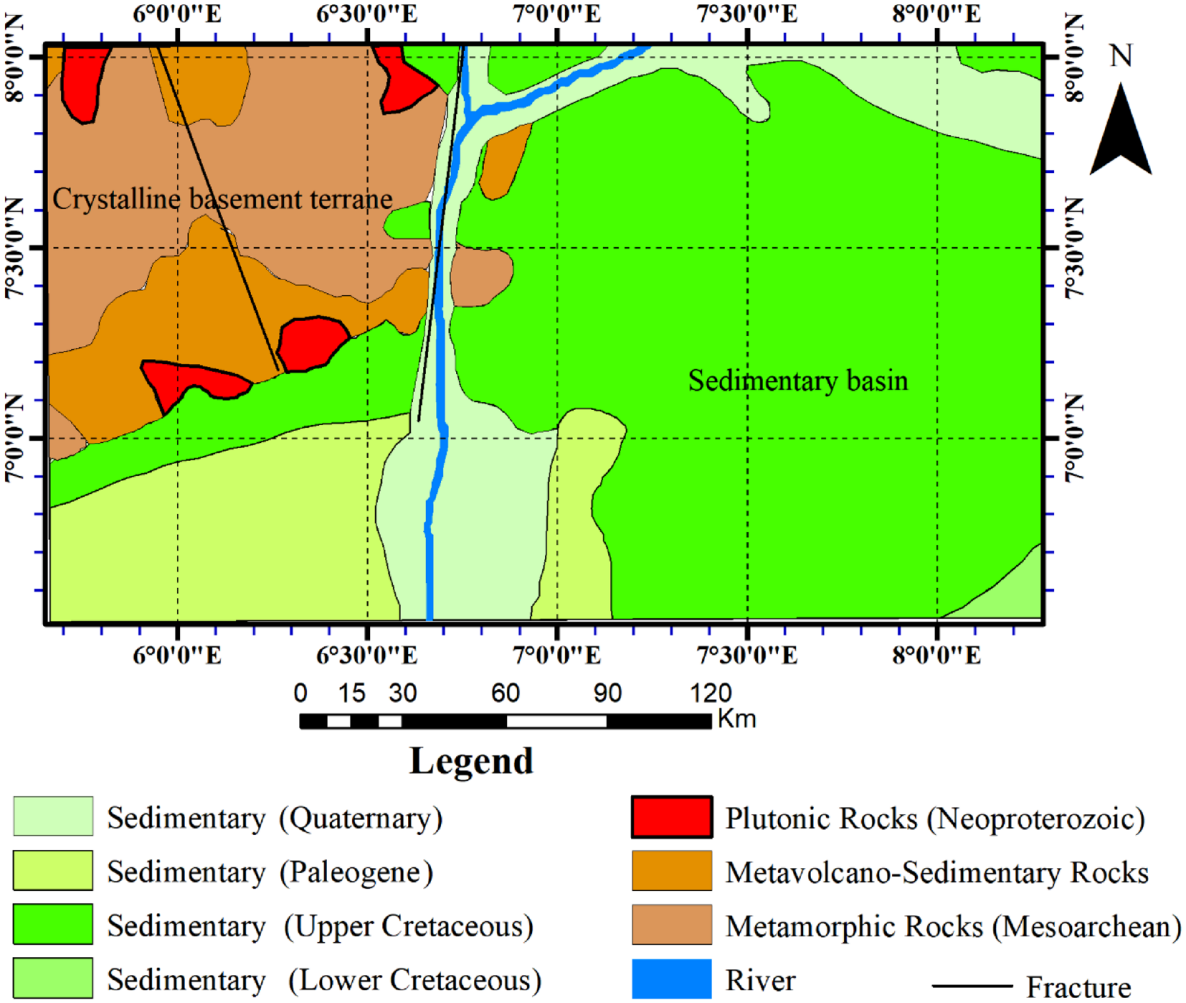
Geological map of the study area showing the regions underlain by the sedimentary and crystalline rocks.

The sedimentary basin in the study area is part of the southern Benue trough and a small part of the Niger Delta basin (Fig. 3). It is underlain, essentially, by the Cretaceous sediments in the north-eastern segments and by the Tertiary sediments in the southern segments^4,35,48^. However, it is worth mentioning that the Cretaceous sediments in the northeastern segments are specifically Upper Cretaceous, whereas the Tertiary sediments in the southern segments range from Tertiary to Quaternary. The part of the study area adjacent to the coastline has an extensive area of Quaternary sediments that continue inland along the Niger-Benue River. The sedimentary sequences in the study area were intruded by mafic igneous rocks belonging to the Tertiary or later^9,35,42,48^.

### Crystalline terrane

The crystalline basement in the study area displays a polycyclic sequence of reactivated Archaean basement comprising variably and extensively migmatized gneisses, orthogneisses, paragneisses, fragments of silicified quartzite-schist belts, and Pan-African granitoid (Fig. 3). The crystalline basement has several regional fractures cutting through it, most of which exhibit NE-SW and NNE-SSW structural alignments. The Kalangai-Zungeru-Ifewara and Anka-Yauri-Iseyin shear zones are notable examples of such regional fractures. Incidentally, a few of the regional fractures, particularly transcurrent faults extend to the South Atlantic Ocean, where they connect with the Atlantic ridge system^49–51^.

### Cretaceous and tectonic evolution of the Benue Trough

**Figure 4:**
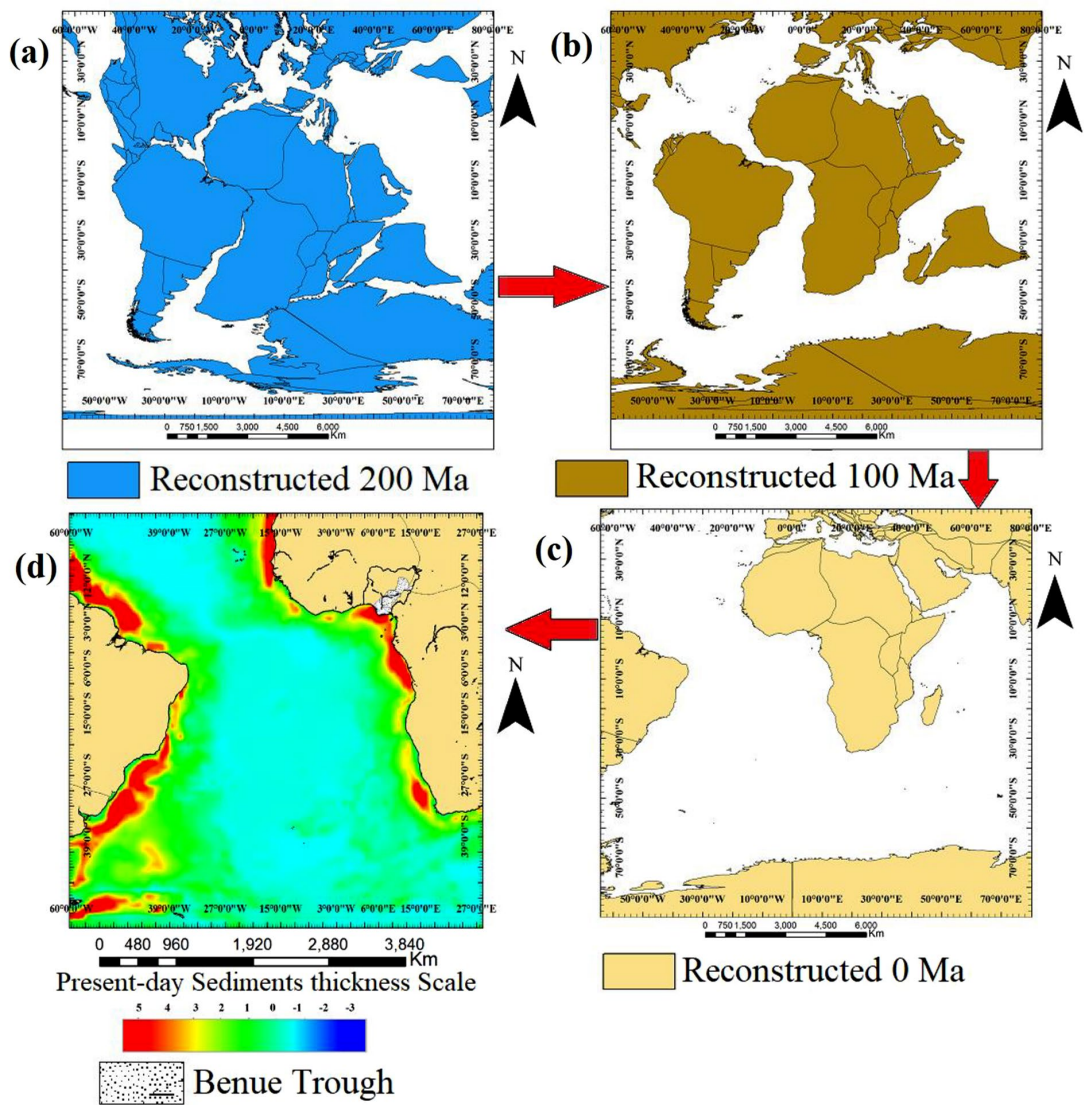
Reconstructed maps showing (**a**) the pre-drift stage of Gondwana (**b**) the early-drift stage (**c**) the post-drift stage (**d**) the opened Atlantic Ocean and the present-day continental sediments thickness.

The Nigerian Benue trough is an aborted subset of a triple junction at the time of the opening of the Gulf of Guinea^1–3,52,53^. The trough formation was initiated by the rifting of the African and South American continental plates, which began in the Cretaceous period^6,9,54,55^. To understand the development of the trough in relation to plate movements, we present a pre-drift reconstruction of Gondwana at 200 Ma which corresponds to the early Jurassic (Fig. 4a)^7^. The map shows the initial position of the African and South American plates. The continents in the southern hemisphere originally existed as one supercontinent at this period^56–60^. However, during the Lower Cretaceous, at about 100 Ma, the seafloor began to spread and the supercontinent split, causing the African and South American plates to drift apart. This led to the opening of the South Atlantic Ocean (Fig. 4b)^7^. The drifting of the continents resulted in continental volcanic activity, earthquakes, and the development of regional continental fault systems^9,61–64^. During this period, the Nigerian Benue trough, aided by the regional intra-sedimentary fault system, began to open which was later aborted. The opened Benue trough experienced marine transgression which led to the deposition of both continental and marine sediments. This was followed by the reactivation of the intra-sedimentary fault system that resulted in major structural deformation characterized by the development of synclinal and anticlinal folds and the subsequent injection of igneous rocks into the basement and the sedimentary sequences^6,9^. Figure 4c shows the present-day position of the African and South American plates which corresponds to the Holocene (0 Ma). The present-day coastline and intra-continental sedimentary thickness across the South Atlantic Ocean are equally shown in Fig. 4d^7,8^.

### Data

#### Magnetic data

**Figure 5:**
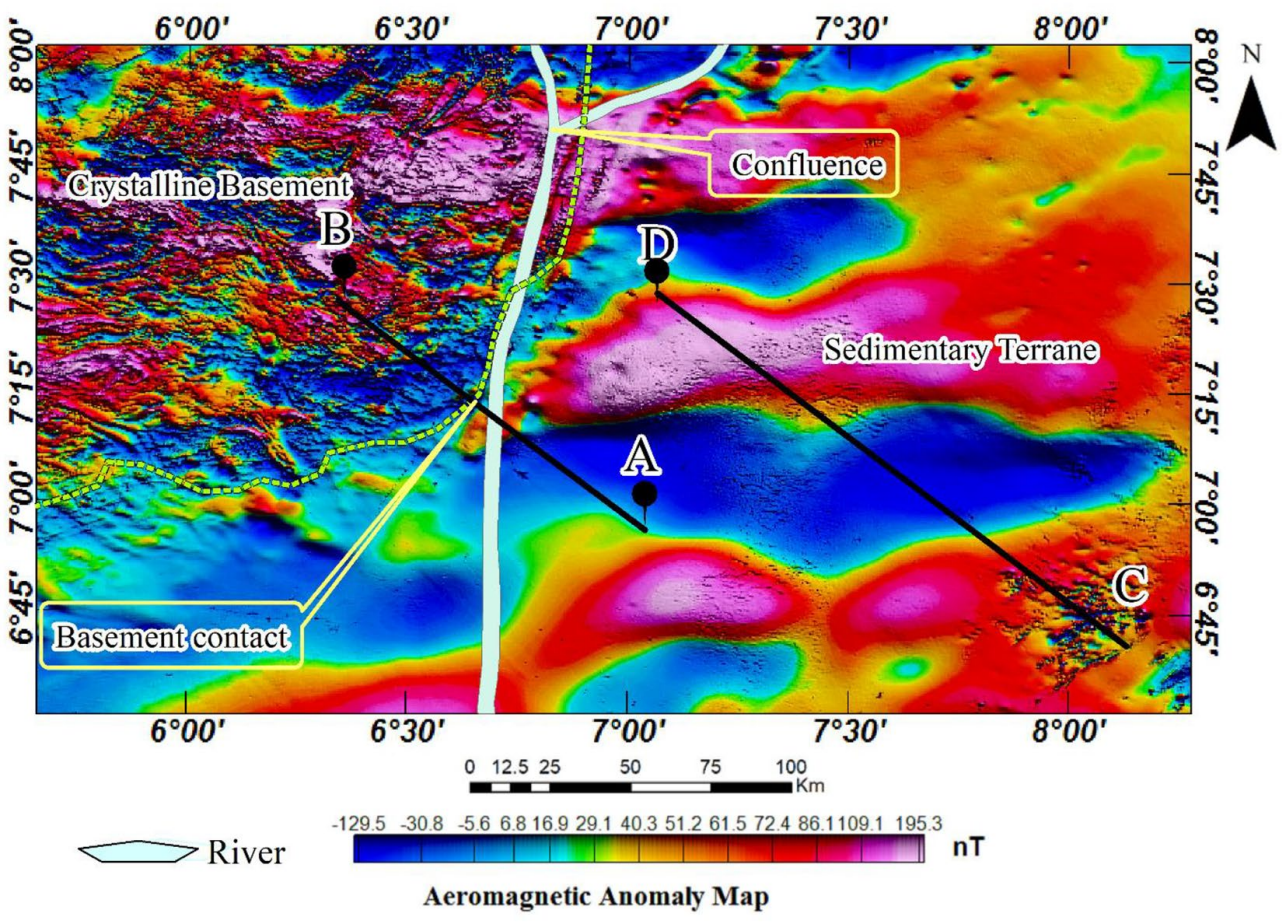
Residual aeromagnetic anomaly map of the study area showing NW–SE profiles.

High-resolution aeromagnetic anomaly datasets across the study area were procured from the Nigerian Geological Survey Agency (NGSA). The acquisition of the unprocessed data was done by Fugro Airborne Surveys company along NW–SE profiles at a magnetic data recording interval of 0.1 s and a mean barometric flight altitude of 80 m^65^. The data were acquired at 500 m profile spacings and 2000 m tie-lines^6^. Essential corrections and data improvements including the removal of temporal variations and the international Geomagnetic reference field (IGRF) were carried out by Fugro Airborne Surveys company^65,66^. Following the elimination of the temporal fluctuations and the IGRF, the residual dataset, representing the magnetic field from the local geological features, underwent gridding at 100 m intervals using the Minimum Curvature technique^65,67^ and presented as a 2-D map (Fig. 5).

The analysis and description of low and high-amplitude magnetic areas reveal distinctive features in the crystalline basement adjacent to the Benue trough. This area exhibits a complex distribution of short-wavelength anomalies. The boundary of the Cretaceous basin and the crystalline basement is generally discernible through a series of magnetic lows, except in the northern segment where a high-amplitude magnetic anomaly extends from the crystalline basement into the Cretaceous basin. Furthermore, within the Cretaceous basin, magnetic lows and highs form alternating belts of long-wavelength anomalies. These are not continuous, instead, they manifest as elongated, separate anomalies. To further distinguish between the two geological terranes, the sedimentary-basement contact was demarcated with a greenish-yellow line (Fig. 5). Furthermore, two profiles A-B and C-D were taken, each stretching 76 km and 147 km across notable parts of the study area. The interpretation of these profiles was based on 2-D structural and stratigraphic analysis.

## Methods

### Total gradient magnitude analysis

Total gradient magnitude (TGM) is one of the edge detection techniques used for interpreting potential field data^68–72^. It is one of the qualitative techniques for interpreting potential field data for delineating geologic structures and rocks. It works by forming maxima directly on the edges of the causal bodies^68–72^. The tool was applied to the aeromagnetic anomaly data of the study area to detect the main geological features and structural alignment which are mainly two-dimensional. The non-dependence of the method on the magnetization direction, remanent magnetization, dip angle, and depths to the top and bottom of sources for two-dimensional magnetic bodies made it an excellent tool for the study area^73,74^. Details of the TGM are available in the published work of^68–72^. The expression for TGM interpretation for gridded magnetic data is shown in Eq. (1).

1 $$|TGM \left(x,y\right)|=\sqrt{{\left(\frac{\partial F}{\partial x}\right)}^{2}+{\left(\frac{\partial F}{\partial y}\right)}^{2}+{\left(\frac{\partial F}{\partial z}\right)}^{2},}$$

where $\frac{\partial F}{\partial x}$ and $\frac{\partial F}{\partial y}$ are the first-order partial derivatives of the residual magnetic intensity field $F$ in $x$ and $y$ horizontal directions respectively while $\frac{\partial F}{\partial z}$ is the first-order partial derivative of the residual magnetic intensity field $F$ in a $z$ vertical direction. The aeromagnetic data of the study area were acquired such that, the profile spacing is sufficiently greater than the station distance, that is $y\gg x$, such that the $\underset{y \to \mathit{ \infty }}{\mathrm{lim}}\left(\frac{\partial F}{\partial y}\right)=0$. Therefore, Eq. (1) reduces to Eq. (2) for delineating two-dimensional geological features^75,76^.

2 $$\left|TGM(x,y)\right|=\sqrt{{\left(\frac{\partial F}{\partial x}\right)}^{2}+{\left(\frac{\partial F}{\partial z}\right)}^{2}.}$$

### Pseudo gravity transformation

A pseudo-gravity is an assumed gravity anomaly observable when a magnetization contrast is replaced by an equivalence density contrast^77–81^. Due to the dipolar nature of magnetic sources and the fact that the magnetization of a body can point in any direction, magnetic responses are far more complicated than gravitational ones. Pseudo-gravity transformation is an anomaly simplifier, making the analysis of magnetic data considerably simpler. The transformation may be a helpful tool for interpreting magnetic anomalies, not because a mass distribution exactly corresponds to the magnetic distribution beneath the magnetic survey, but rather because gravity anomalies are sometimes more instructive and simpler to analyse than magnetic anomalies^80–82^. By converting the magnetic anomaly to a pseudo-gravity anomaly and examining the anomaly for maximum horizontal gradients, it is possible to make use of the fact that gravity anomalies over tabular bodies have the steepest horizontal gradients roughly over the edges of the bodies^75,80,81,83^. Furthermore, pseudo-gravity transformation can potentially reduce the stronger effect of shallow magnetic sources and increase the dominance of magnetic anomalies from deeper sources (Panepinto et al.^81^). The relationship between the magnetic potential and gravitational field established by Poisson leads to the pseudo-gravity transformation^81^. The magnetic and gravitational scalar potentials of a body with uniform magnetization and density in volume $v$ are given by Eqs. (3) and (4) respectively^77,81^

3 $$V\left(q\right)=-M{\nabla }_{q} {\iiint }_{V}\frac{1}{r}dv,$$

4 $$U\left(q\right)=G\rho {\iiint }_{V}\frac{1}{r}dv,$$

where $q$ is the data observation point, $r$ is the source distance, $M$ is the magnetization distribution, $G$ is the gravitational constant and $\rho$ the source density. Using Eqs. (3) and (4) we obtained Eqs. (5), (6) and (7):

5 $$V\left(q\right)=-\frac{1}{G\rho }M{\nabla }_{q} U,$$

where

6 $${g}_{M}={\nabla }_{q} U,$$

7 $$V\left(q\right)=-\frac{1}{G\rho }M{g}_{M}.$$

From Poisson’s relation, ${g}_{M}$ refers to the component of gravity in the magnetization direction. The pseudo-gravity operator converts the magnetic anomaly into a gravity-like response as if the body’s magnetism were replaced with the same density distribution, where density and magnetic susceptibility have a perfect linear association^77,81,82^. The pseudo-gravity transformation was applied to the aeromagnetic anomaly data in the current study area to simplify the magnetic anomaly and, where necessary, to locate magnetically strong and magnetically dense geologic bodies within the Cretaceous basin. Since the pseudo-gravity map is an equivalent gravity emanating only from magnetic sources and because the main sources of strong magnetic anomalies in the basin are the igneous intrusions and the basement, the pseudo-gravity analysis was found suitable for these purposes. Further details regarding the theory underlying the pseudo-gravity integration are available in the published work of Ref.^77,78,81^.

### Tilt derivative analysis

The tilt derivative (TDR) of a potential field data computes the arctan of the ratio of the potential field’s first-order vertical derivative to its horizontal gradient magnitude^84^. The tool works by enhancing magnetically quiet sources that were previously concealed by high-amplitude magnetic sources. It is suitable for delineating magnetic minerals hosted for example by a more magnetic mafic igneous rock^74^. The TDR, however, is very sensitive to the geomagnetic inclination and declination^73^. Further details of the TDR method are contained in the published work of Ref.^84^. For this study, the dependence of TDR on geomagnetic inclination and declination was resolved by transforming the aeromagnetic data to a pseudo-gravity map before applying it^85^. The TDR, according to Ref.^84^ is defined as shown in Eq. (8).

8 $$TDR={tan}^{-1}\frac{FVD}{\left|HGM(x,y)\right|}.$$

Equations (9) and (10) defined each of the $FVD$ and $\left|HGM\left(x,y\right)\right|$

9 $$FVD=\frac{\partial F}{\partial z},$$

10 $$\left|HGM(x,y)\right|=\sqrt{{\left(\frac{\partial F}{\partial x}\right)}^{2}+{\left(\frac{\partial F}{\partial y}\right)}^{2}},$$

where $FVD$ and $\left|HGM(x,y)\right|$ are the first-order vertical derivative and horizontal gradient magnitude of the aeromagnetic anomaly data respectively.

### Source parameter imaging analysis

Reference^86^ introduced the source parameter imaging (SPI) technique. The method is based on a complex analytic signal that calculates source parameters from gridded aeromagnetic data. It is also known as the local wavenumber technique. The tool when applied to potential field data formed peaks over isolated contacts. The estimation of the depths is carried out without making assumptions about the source thickness^86^. The map is more similar to geology than the magnetic map or its derivatives^86,87^. The SPI solution grids contain information about the edge locations, depths, dips, and susceptibility contrasts. The local wavenumber in Eq. (11) was employed in the estimation of the depths in the source parameter imaging (SPI) method^86,87^.

11 $$K=\frac{\frac{{\partial }^{2}{\varvec{F}}}{\partial {\varvec{x}}\partial {\varvec{z}}}\frac{\partial {\varvec{F}}}{\partial {\varvec{x}}}\boldsymbol{ }+\boldsymbol{ }\frac{{\partial }^{2}{\varvec{F}}}{\partial {\varvec{y}}\partial {\varvec{z}}}\frac{\partial {\varvec{F}}}{\partial {\varvec{y}}}\boldsymbol{ }+\boldsymbol{ }\frac{{\partial }^{2}{\varvec{F}}}{\partial {{\varvec{z}}}^{2}}\frac{\partial {\varvec{F}}}{\partial {\varvec{z}}}}{{\left(\frac{\partial {\varvec{F}}}{\partial {\varvec{x}}}\right)}^{2}+\boldsymbol{ }{\left(\frac{\partial {\varvec{F}}}{\partial {\varvec{y}}}\right)}^{2}+{\boldsymbol{ }\left(\frac{\partial {\varvec{F}}}{\partial {\varvec{z}}}\right)}^{2}},$$

where K is the local wavenumber. The depth estimates are thus calculated using the reciprocal of the local wavenumber given in Eq. (12).

12 $$Dept{h}_{x=0}=\frac{1}{{K}_{max}}.$$

K_max_ is the maximum value of K above the source edge.

## Results and discussion

### Total gradient magnitude anomaly map

**Figure 6:**
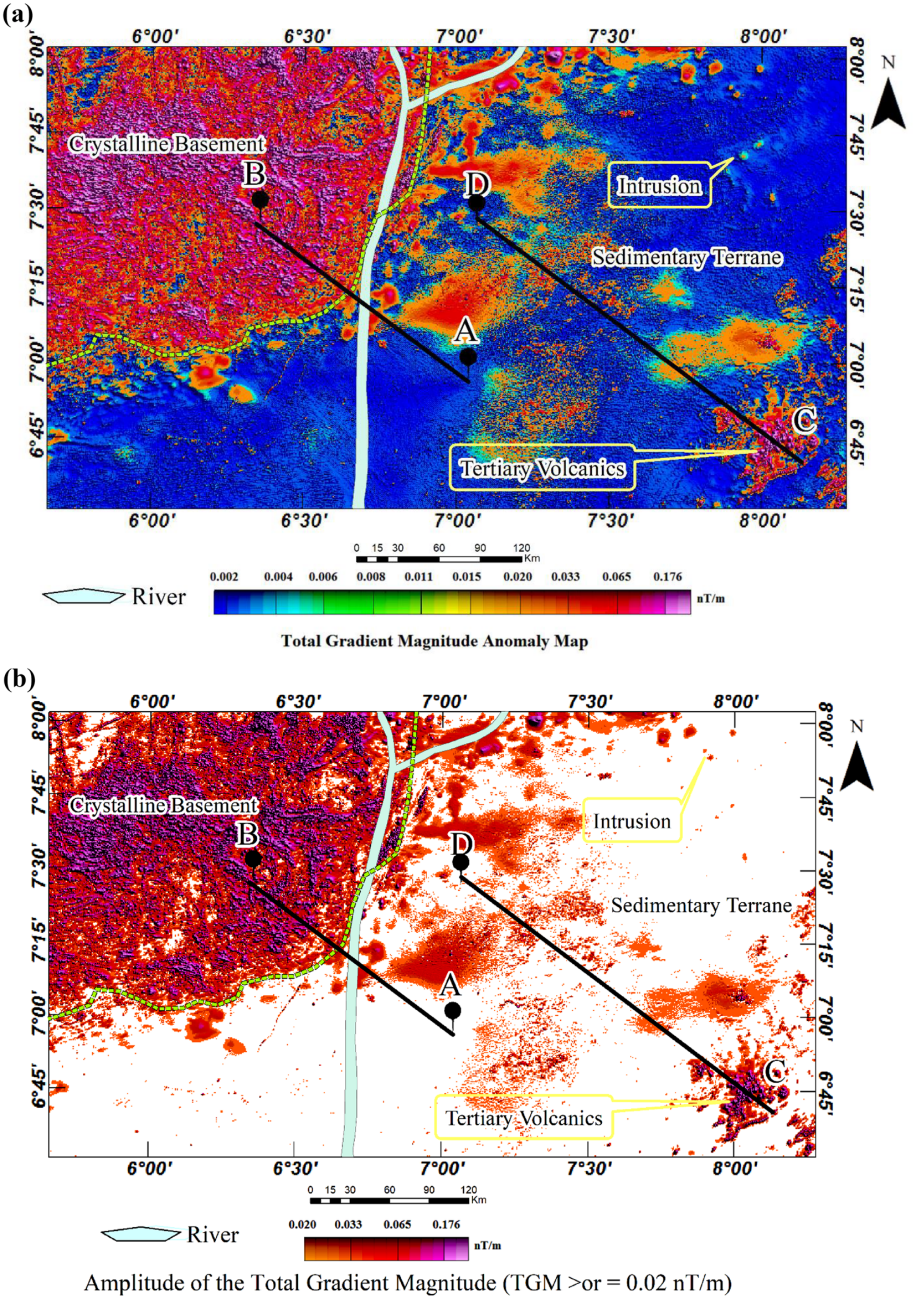

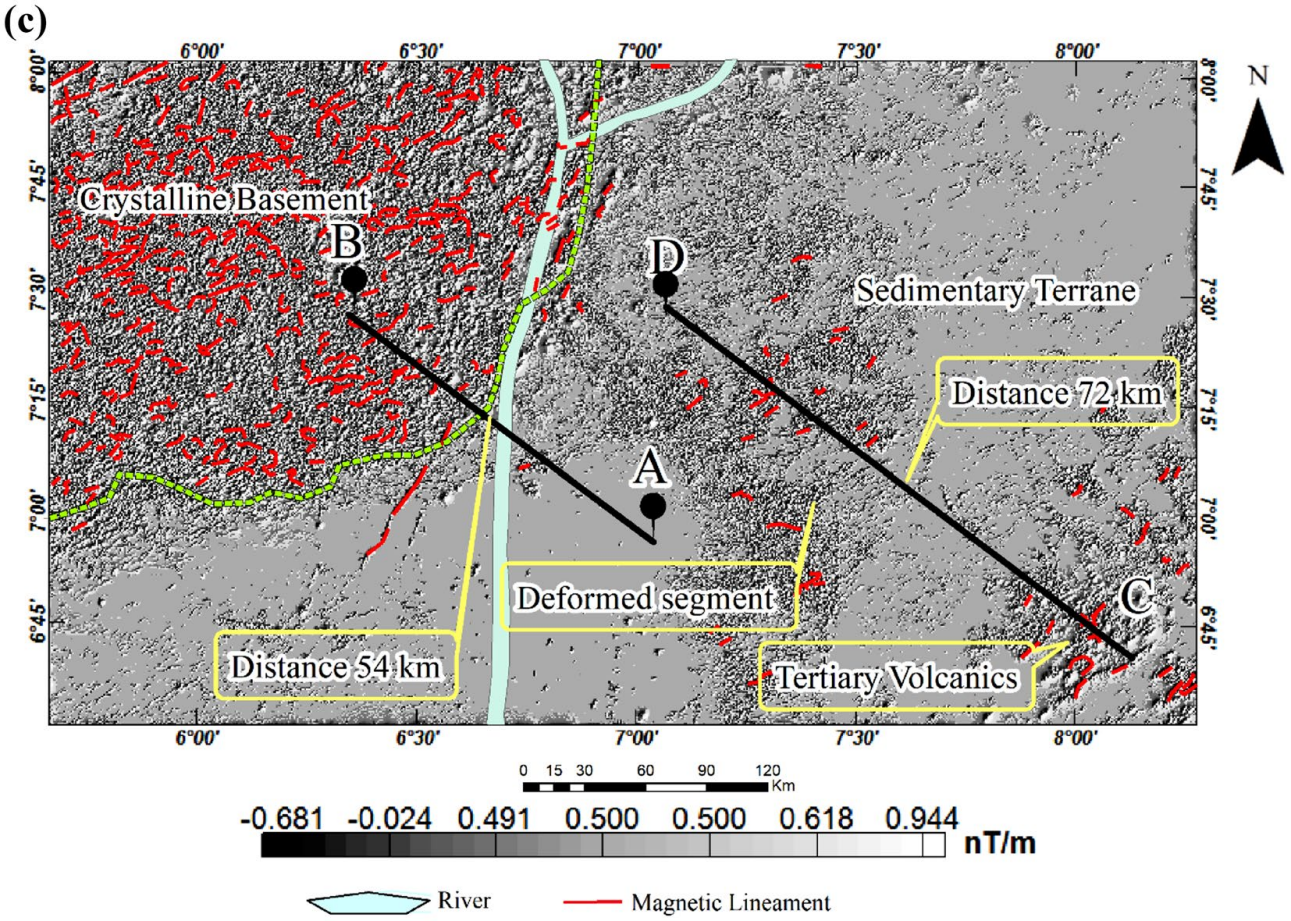
(**a**) Total gradient magnitude (TGM) aeromagnetic anomaly map of the study area. (**b**) Total gradient magnitude (TGM) amplitude map of the study area. (**c**) Hill-shaded total gradient magnitude (TGM) with magnetic lineaments.

The total gradient magnitude (TGM) analysis was applied to the aeromagnetic data to enhance short-wavelength aeromagnetic anomalies corresponding to shallow-seated geological structures and igneous intrusions. The result reveals a significant number of shallow-seated linear and isolation geological features that can be interpreted as fractures and igneous intrusions respectively (Fig. 6a). The fact that the study area extends beyond the Cretaceous basin into the crystalline basement, the TGM analysis delineates distinct structural differences between these two geologically different environments, identifying their boundary (depicted by yellow broken lines). Notably, the crystalline basement displays extremely complex structural architecture with most parts exhibiting imprints of shallow-seated felsic to intermediate igneous rocks (Fig. 6b). These rocks overwhelmingly conceal the metamorphic basement, resulting in shallow overburden thicknesses throughout the crystalline basement^88,89^. The crystalline basement equally displays complex structural orientation, probably as a result of the overprinting of multiple orogenic structures (Fig. 6c)^90–92^.

In contrast, the Cretaceous basin shows distinctive igneous rock distribution, enabling the classification of the basin into two separate regions. The first region contains a few igneous rocks, representing a fairly intruded area. This is notable in the northeastern and southwestern parts of the study area (Fig. 6b). The second contains several igneous rocks, representing a highly intruded region. The southeastern part and particularly most areas around the sedimentary-basement contact are notable for a high degree of igneous intrusions. The margin of the Benue trough and the crystalline basement in southwestern parts have been identified with indiscriminate artisanal gold mining^93^. The occurrence of gold in this area could potentially be linked to the prevalence of igneous intrusions. In this case, the contact between these intrusions and high-grade metamorphic rocks, such as quartz-schist, quartzite, and gneisses, could serve as possible locations to maximise the exploitation of gold.

### Pseudo-gravity transformation anomaly map

**Figure 7:**
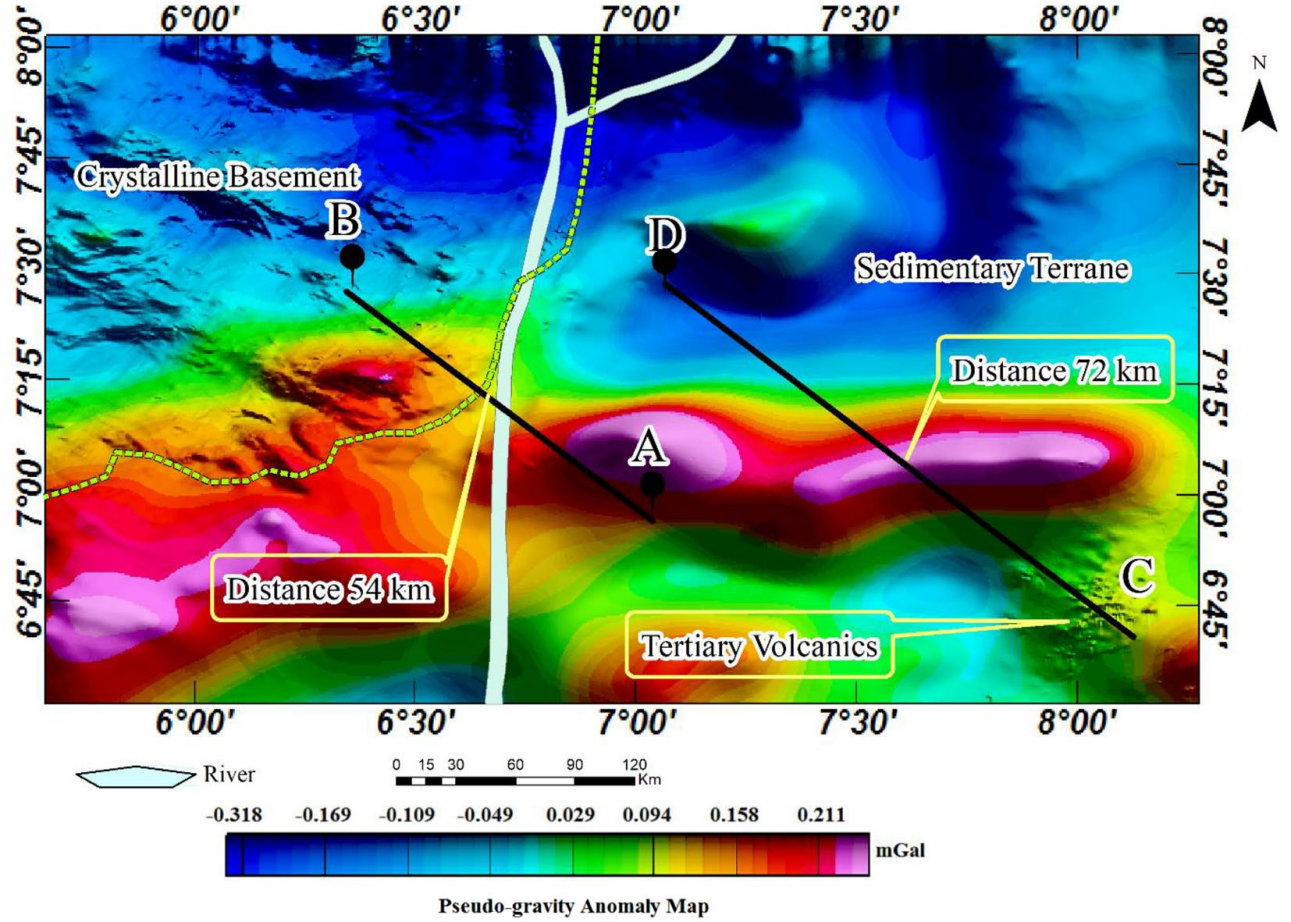
Colour-shaded pseudo-gravity anomaly map of the study area.

To reveal magnetically strong geological formations underlying the Cretaceous basin, we applied a pseudo-gravity transformation (PGT) to the aeromagnetic anomaly map. The result shows an elongated region of the basin characterized by a high pseudo-gravity anomaly that stretches eastward in parts of the southern study area (Fig. 7). This region has positive pseudo-gravity anomalies that coincide with the centre of the source and is flanked on both sides by linear negative anomalies. This pattern is common in rift valleys and indicates possible crustal thinning in regions with negative pseudo-gravity anomalies, along with crustal thickening in areas displaying positive pseudo-gravity anomalies^3,5,33,94–97^. By comparing the pseudo-gravity anomalies originating from this magnetic body with those from the crystalline basement and other parts of the basin, we observe a strong magnetic signature, suggesting the existence of a magnetically dense and highly deformed segment. It is, therefore, possible that the deformed segments are mainly of mafic composition, resulting in highly dense and magnetically strong features. The pseudo-gravity result in this study is therefore significant because it demonstrated that, despite recent changes, the structures imprinted during the Cretaceous are well-preserved.

### Tilt-derivative anomaly map

**Figure 8:**
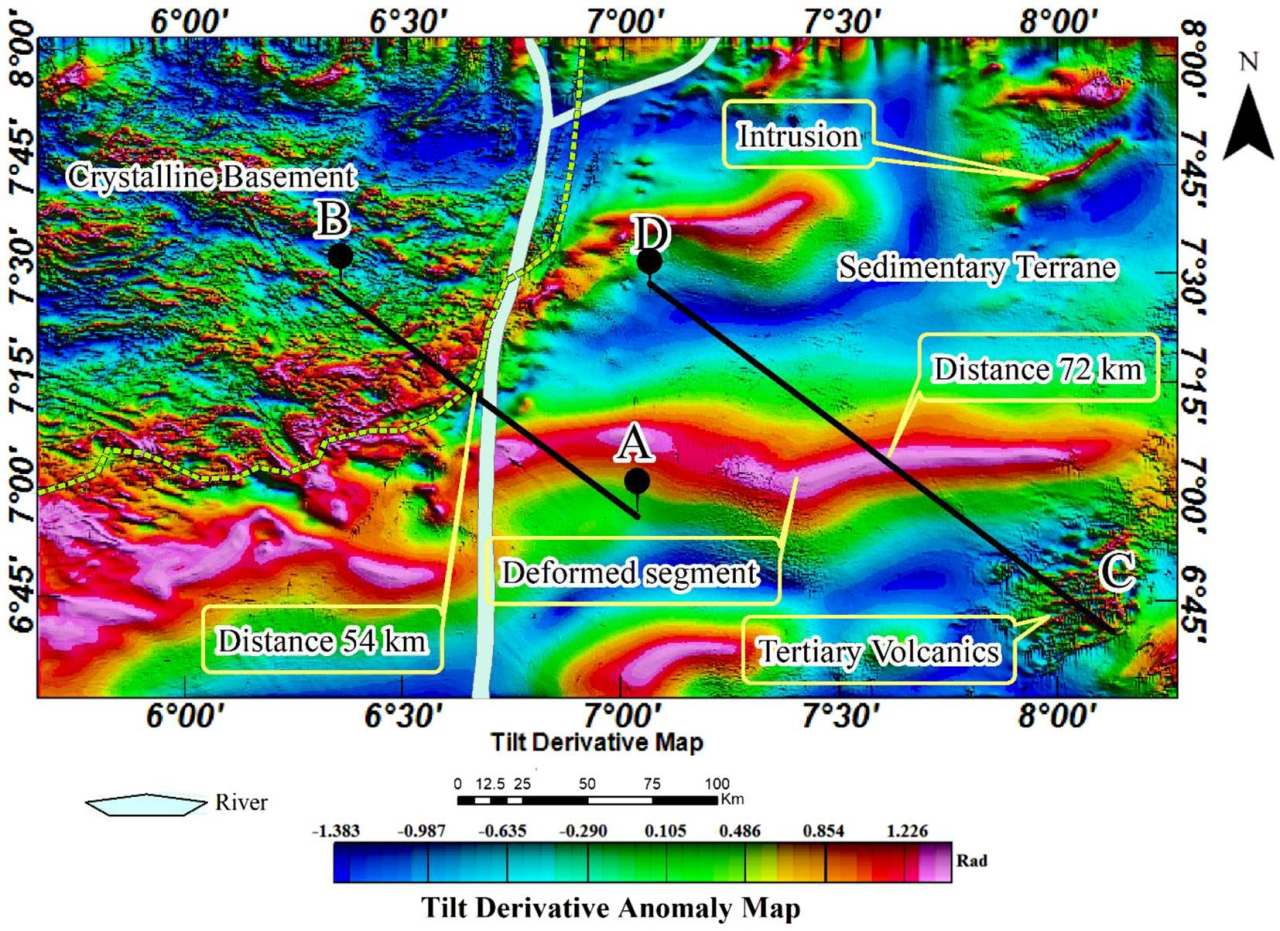
Tilt derivative of the pseudo-gravity anomaly map.

The tilt-derivative (TDR) map (Fig. 8) was derived from the pseudo-gravity anomaly map to enhance subtle magnetic features within highly magnetic mafic igneous rocks (see Fig. 7). It is important to understand that TDR is sensitive to the direction of magnetization. It was, therefore, applied to an aeromagnetic dataset that has undergone a pseudo-gravity transformation to limit its dependency. Reports indicate the presence of lead-zinc-copper mineralization linked with igneous rocks, specifically diorite in addition to the structurally-controlled hydrothermal mineralization in many parts of the southern Benue trough^9,34,39,98–101^. However, the diamagnetic nature of these metals poses challenges in detecting them within the host rocks, particularly mafic igneous rocks. The ability of the TDR to enhance subtle magnetic anomalies, while simultaneously suppressing strong magnetic anomalies was found suitable for identifying potential locations for follow-up mineral investigation. Ultimately, the result identified possible locations to focus on a detailed investigation of the minerals. The result further revealed the dyke intrusions within the northeastern study area, originally identified in the TGM map (see Fig. 6a and b). The distinct linear features of these intrusions were revealed, subsequently highlighting the mineralized segments that were not revealed with other analyses (Fig. 8). This emphasised the possibility of volcanic-hosted lead-zinc-copper mineralization as earlier reported by Ref.^6,9,39^. Generally, the TDR map shows specific parts of the sedimentary basin that may be suitable for a follow-up mineral investigation in the study area. It equally highlights the margin of the Cretaceous basin adjoining the crystalline basement which further substantiates the observation that the region can be a good location for a follow-up investigation of gold mineralisation. A notable drawback associated with TDR applied to pseudo-gravity anomalies is the potential for edge deformation, which could be misinterpreted as skew in the original data.

### Two-dimensional interpretation of the SPI analysis

The igneous rocks in the study area are not exposed. This made direct measurement of their susceptibility impossible. Similarly, we could not identify any paleomagnetic reports in the area, so no information about the remanent magnetization and rock magnetic polarity is available. We, therefore, relied on the source parameter imaging (SPI) for the 2-D interpretation of the subsurface morphology. This is because the estimation of source depth and, ultimately, the display of 2-D subsurface morphology are independent of the source magnetic polarity, remanent magnetization, dip, and strike^87^.

**Figure 9:**
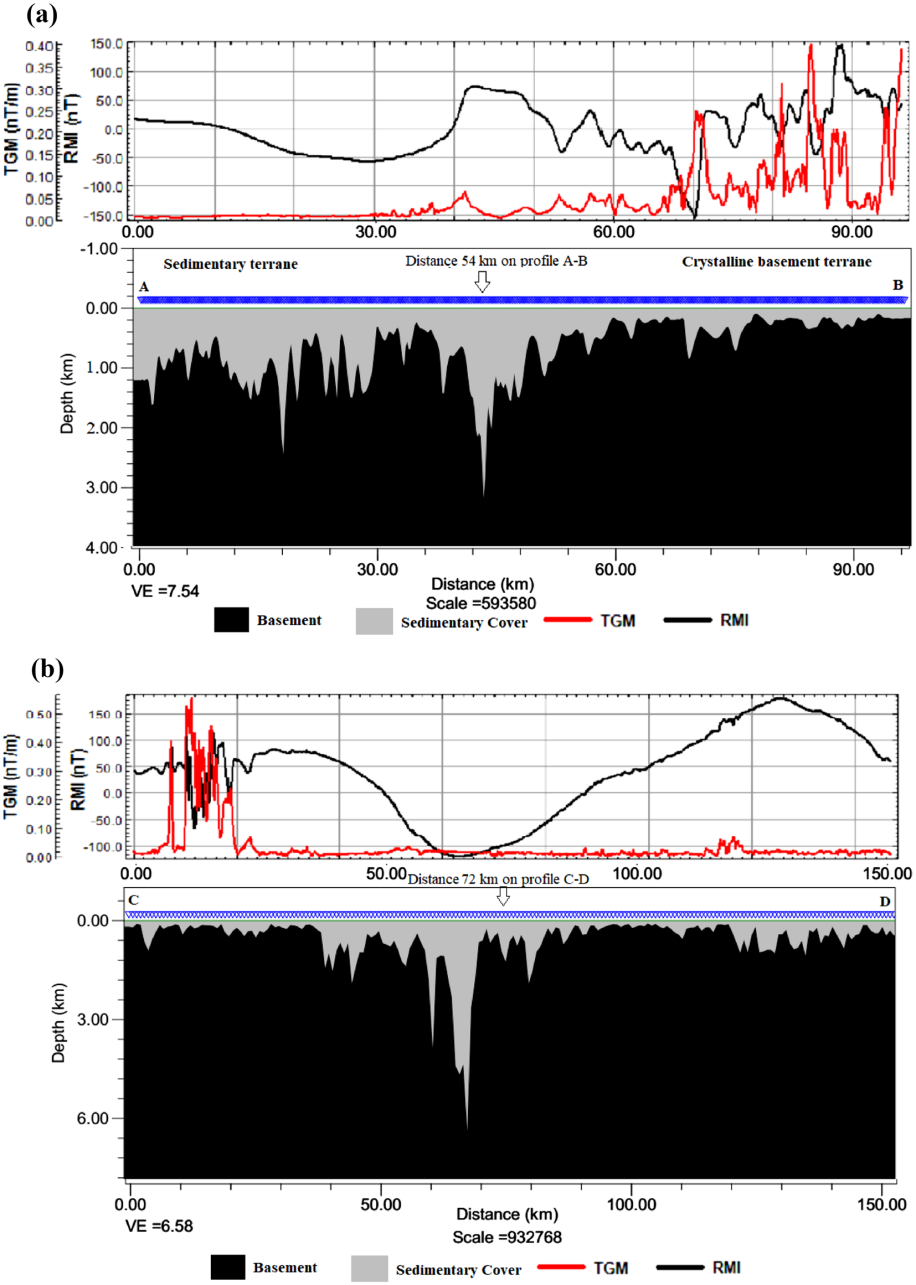
Maps showing 2-D subsurface morphology across profiles (**a**) A-B. (**b**) C-D.

To understand the subsurface morphology, we present two SPI 2-D models from the aeromagnetic profiles A-B and C-D stretching 76 km and 147 km respectively across the study area. This analysis was implemented using magnetic inclination, declination and total magnetic field intensity of ${-10.57}^{0}$, ${-2.41}^{0}$ and $33069.47 nT$ respectively. The source parameters namely analytic signal amplitude, local phase, local frequency, local wavenumber and, ultimately, the source depths were estimated. The results, particularly the 2-D model A-B show the basement to be intensely fragmented and variably subsided within the basin (Fig. 9a). The floor of the basin is irregularly deformed leading to a considerable variation in the thicknesses of the sedimentary basin from place to place. The irregular floor is most likely the result of an intense block faulting, which began at the time of the formation of the trough as a ‘failed arm’ of a triple junction (an aulacogen) when the Africa and South America plates drifted apart during the Cretaceous (see Fig. 4). The complex deformation of the basement is revealed in the SPI 2-D model C-D (Fig. 9b). The elongated segment (see distance 72 km in Figs. 7 and 8) was revealed in the 2-D model to have experienced an upward displacement (Fig. 9b). This segment is flanked on both sides by regions displaying downward displacements as initially revealed in the pseudo-gravity result (see Fig. 7). As a result, a shallow sedimentary thickness of 100 m or less is observed over the parts of the basement that were displaced upward and up to 3 km for the areas displaced downward. The deepest sedimentary piles, especially where the basement is folded downward represent possible traps for hydrocarbon given that other conditions are favourable. The margin of the sedimentary and crystalline basement terranes also shows intense basement subsidence that extends downward to about 3.5 km. Furthermore, the sedimentary-basement boundary located at 54 km on profile A-B (Figs. 7 and 8), exhibits an abrupt downward displacement of the basement to a depth of up to 3.5 km (Fig. 9a). This area likely formed during the initiation of the southern Benue trough as a consequence of continental crust rifting. Beyond the sedimentary terrane in the crystalline basement terrane, the basement is fairly deformed resulting in extremely thin overburden or younger sediments (Fig. 9a).

### Structural map

**Figure 10:**
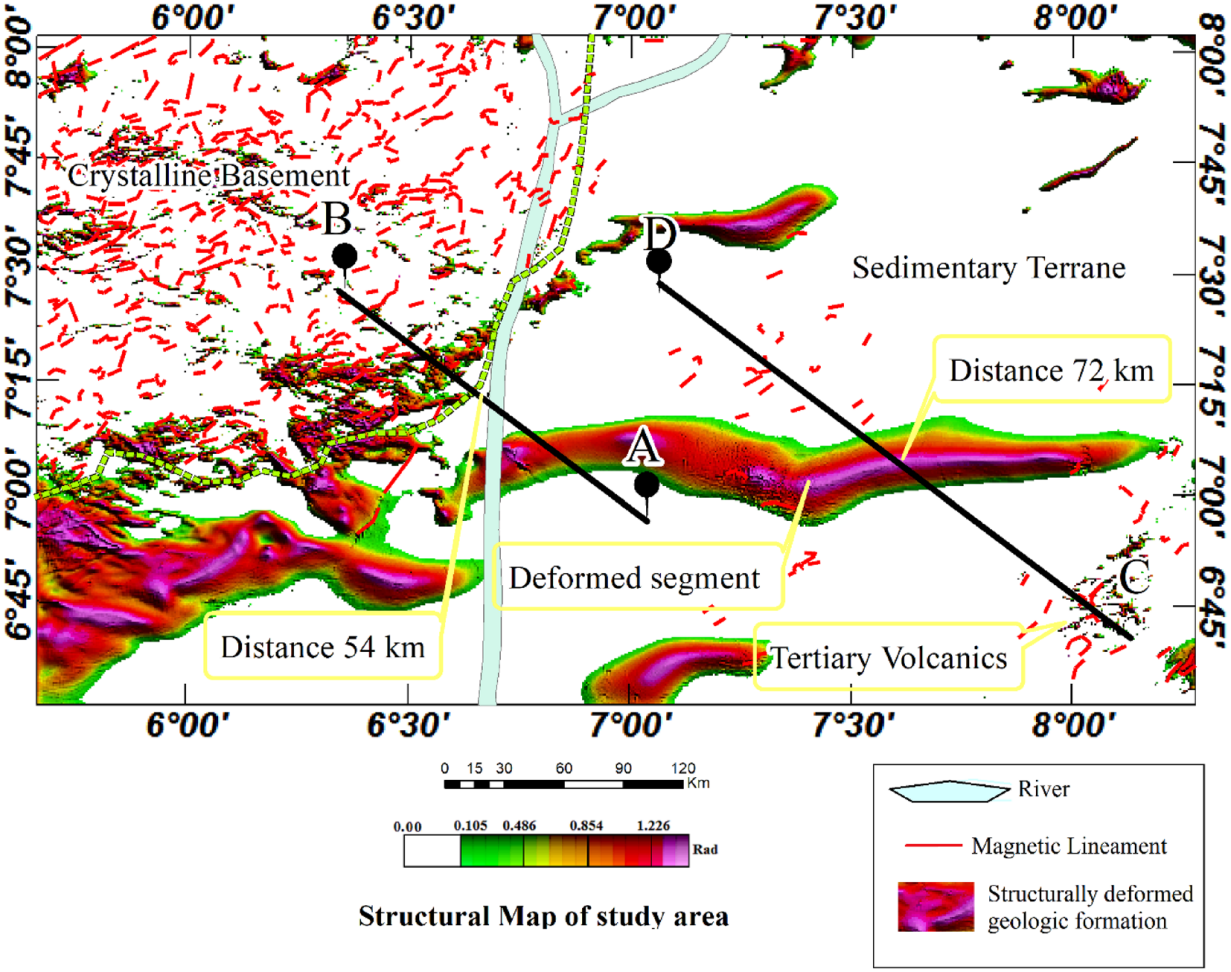
Structural map of the study area.

The application of various edge detection techniques to the aeromagnetic anomaly data enhanced different geological structures and igneous bodies within the studied area. The magnetic lineaments were automatically extracted from the amplitudes of the TGM (see Fig. 6) while the structurally deformed elongated formation in the study area was derived from the TDR analysis (see Fig. 8). The map shows geological features in the crystalline basement and the sedimentary basin. Notably, the Cretaceous basin shows only a few lineaments while the crystalline basement is characterised by a densely populated lineament exhibiting complex geological orientation. The structurally deformed segment of the basin, however, exhibits mainly eastward structural alignments. The boundary of the basin and the crystalline basement which is 54 km on the profile A-B represent important discontinuity. The intrusion areas, including the elongated segment within the basin and along its boundary, constitute key areas for detailed investigation aimed at mineral exploitation, particularly for minerals such as gold, lead, zinc, and copper (Fig. 10).

## Conclusion 

The utilization of aeromagnetic data for investigating the crustal structures and igneous bodies within the sedimentary basin of the southern Benue trough and the adjacent crystalline basement of southwestern Nigeria has yielded significant findings. Numerous linear geologic structures, including faults, as well as basement subsidence and volcanic intrusions, have been detected. Notably, a section of the sedimentary basin exhibits substantial upward and downward displacements of the basement, resulting from intense deformation and block faulting. These geological events played a crucial role in shaping and reshaping the entire Benue trough since the Cretaceous period. The crystalline basement in the study area is separated from the sedimentary basin by a large graben that extends as far down as 3 km. Moreover, a substantial igneous body has been identified in the southern part of the sedimentary basin. This igneous body exhibits abnormally high density and strong magnetic properties, characterized by positive pseudo-gravity anomalies. Surrounding the igneous body on both sides are linear negative anomalies, indicating the presence of a rift valley. The occurrence of magnetically quite causative bodies associated with igneous bodies within the sedimentary basin and along the margin of the crystalline basement suggests the need for further geological surveys to identify potential locations for gold, lead, zinc, and copper exploitation. These surveys will be instrumental in determining the specific areas where these valuable mineral resources may be found. Finally, the areas of severe downward displacement with considerable accumulation of marine sediments suggest high-probability zones for follow-up hydrocarbon investigation.

## Data Availability

The reconstruction data analysed during the current study are available in the Earthbite repository, https://www.earthbyte.org/gplates-2-3-software-and-data-sets/. However, the aeromagnetic data that support the findings of this study are available from the Nigerian Geological Survey Agency but restrictions apply to the availability of these data, which were used under license for the current study, and so are not publicly available. The data are however available from the authors upon reasonable request and with permission of the Nigerian Geological Survey Agency.
